# Jeffrey W. Pollard: a tribute

**DOI:** 10.1242/dmm.050325

**Published:** 2023-06-21

**Authors:** Takanori Kitamura

**Affiliations:** MRC Centre for Reproductive Health, Queen's Medical Research Institute, The University of Edinburgh, Edinburgh EH16 4TJ, UK

Jeffrey Pollard, a big star in macrophage research, passed away peacefully on 1 May 2023. The passing of this revered leader and beloved colleague caused deep sadness, and is a huge loss for the scientific community.

**Figure:**
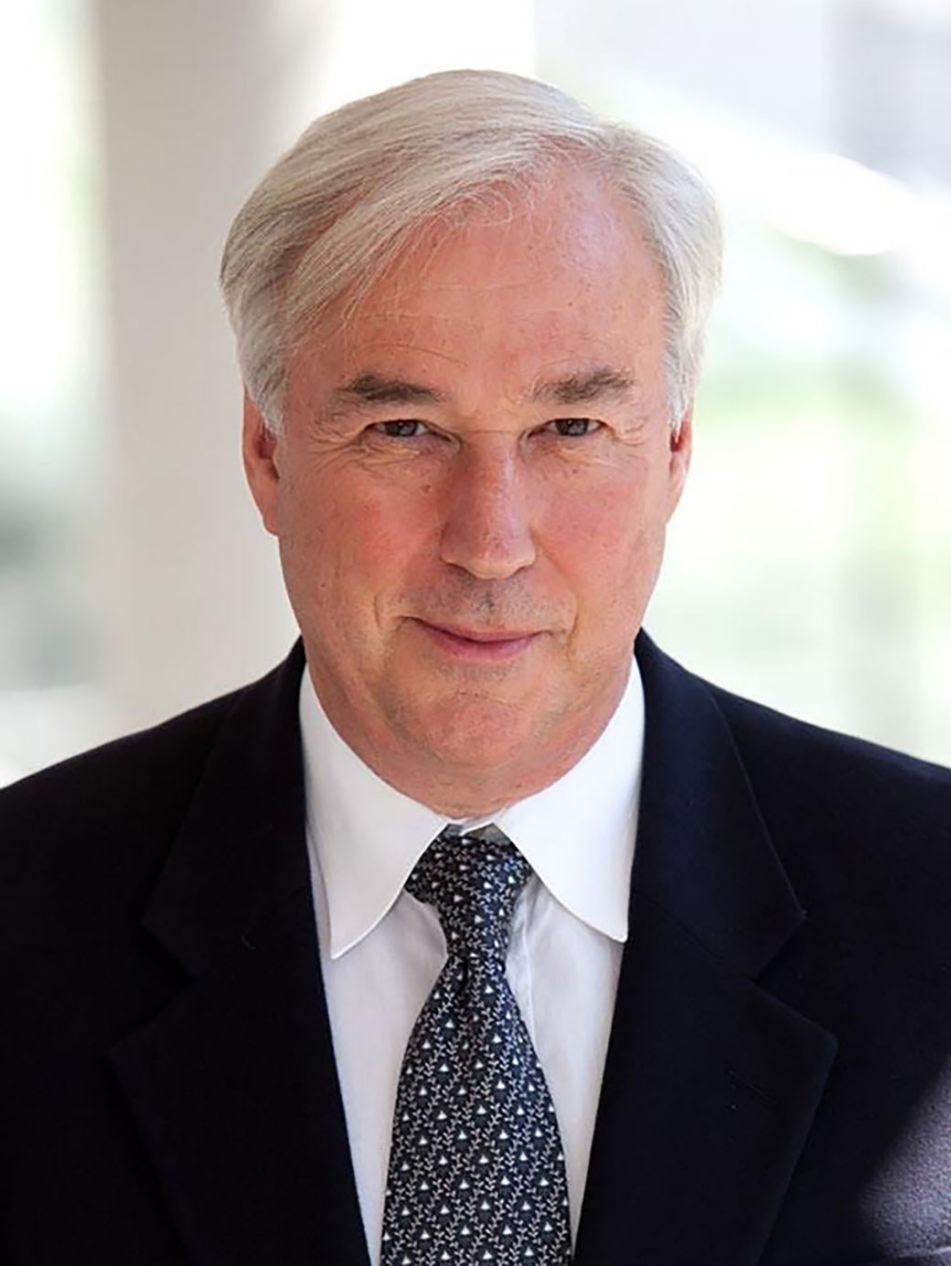


**Figure:**
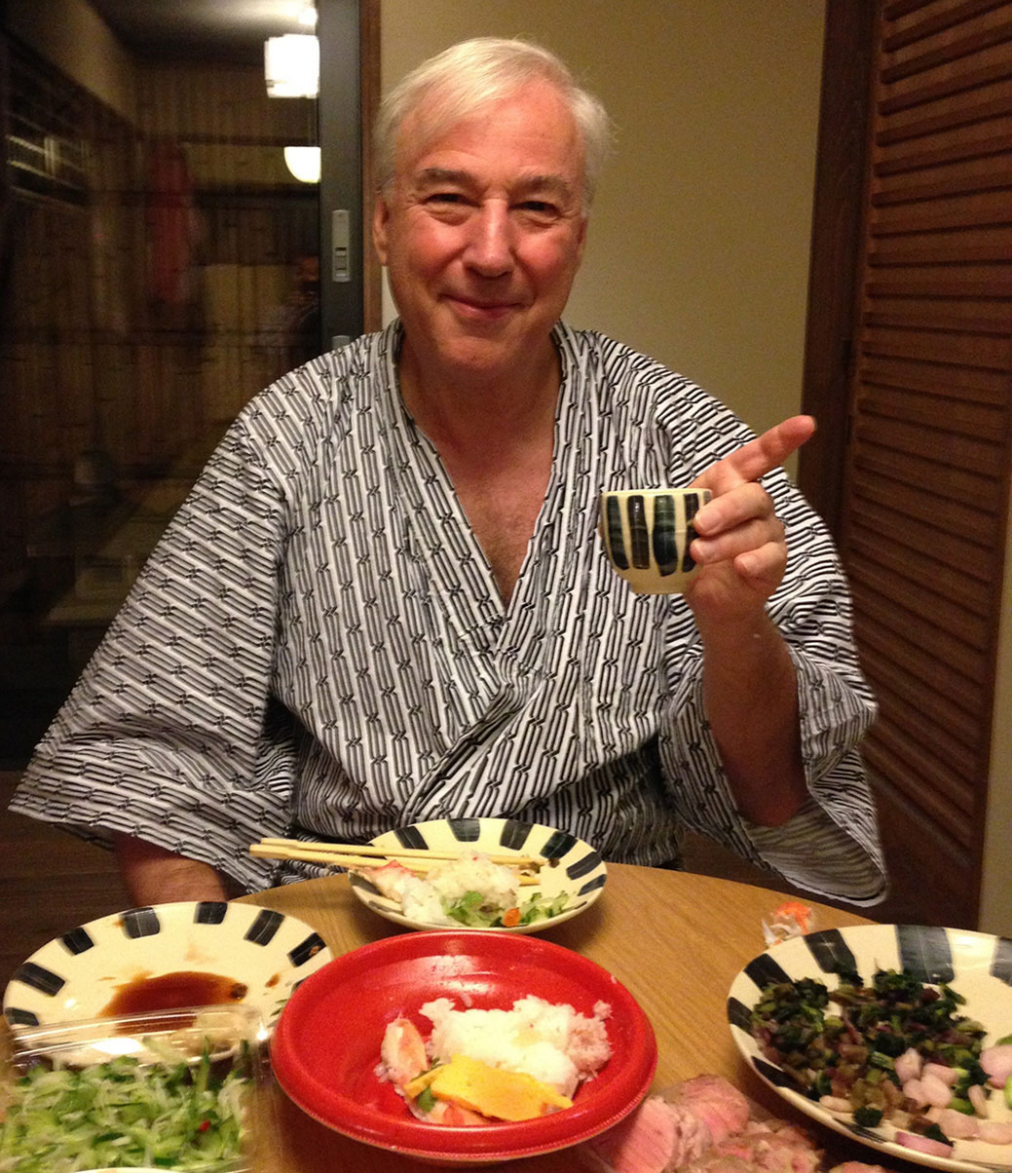


Throughout his professional journey, Jeff enthusiastically conducted extensive research on female reproductive health. His primary focus was on unravelling the intricate mechanisms controlling uterine epithelial cell proliferation through female sex steroid hormones. He also focused on the involvement of macrophages in tissue homeostasis and repair. Moreover, he placed particular emphasis on unravelling the role of macrophages in malignant breast cancer. His outstanding achievements in these areas earned him numerous honours and awards, which include election to fellowships of the American Association for the Advancement of Science, the Royal Society of Biology, the Royal Society of Edinburgh and the Academy of Medical Sciences, a Rothschild Yvette–Meynet Curie Award, the American Cancer Society Medal of Honour for Basic Science Research, the Royal Society Wolfson Research Merit Award and a Wellcome Trust Senior Investigator Award.

Jeff graduated from the University of Sheffield, where he obtained a first-class special honours degree in zoology and earned a PhD at the Imperial Cancer Research Fund in the UK. After spending a post-doctoral period at the Ontario Cancer Institute in Canada, he took a faculty position at King's College University of London. Afterwards, he worked as an assistant professor and then professor at the Albert Einstein College of Medicine (AECOM) in the USA for 24 years. At AECOM, Jeff served concurrently as Director of the Center for the Study of Reproductive Biology and Women's Health, Deputy Director of the Albert Einstein Cancer Center, the Louis Goldstein Swan Chair in Women's Cancer Research, and Director of Specialized Cooperative Centers in Reproduction & Infertility Research.

During such a busy career, he conducted several ground-breaking studies. Initially, Jeff was interested in the roles of cytokines and their receptors in the female reproductive tract, and identified that colony-stimulating factor 1 (CSF-1) signalling is essential for pre-implantation development. He also defined how progesterone negatively regulates the growth of uterine epithelial cells induced by oestrogen. His studies revealed the role of downstream signalling from the hormone receptors through growth factor signalling and the engagement of KLF transcription factors. These studies for the first time linked the actions of sex steroid hormones to the immune system through their regulation of CSF-1, which controls macrophage and trophoblast functions.

Jeff was very proactive in introducing new technology. At a time when genetically modified mice were not as common as they are now, he introduced osteopetrotic (*op/op*) mutant mice into his lab and demonstrated that the absence of CSF-1 causes macrophage deficiency in these mice. Using this model, he demonstrated that macrophages regulate branching morphogenesis of the mammary gland during pregnancy. His research in this area significantly contributed to the establishment of the concept that macrophages are cells with remarkable functional diversity that play roles in immunity, development, homeostasis and tissue repair (expressed as “a cell for all seasons”).

Jeff's extensive knowledge and curiosity pushed his research into a new area – the tumour microenvironment. Using the *op/op* mice and spontaneous breast cancer model mice (MMTV-PyMT mice), he identified that macrophage deficiency suppressed progression of mammary tumours to malignancy. Starting with this landmark discovery, Jeff demonstrated that tumour-associated macrophages (TAMs) play pivotal roles in multiple processes in breast cancer metastasis, that is, angiogenesis, cancer cell invasion and intravasation in the primary site, and extravasation and persistent growth of metastasizing cancer cells in the secondary site.

In 2013, Jeff joined the University of Edinburgh, UK as a Director of the Medical Research Council Centre for Reproductive Health. He continued to study the mechanisms by which macrophages promote tumour progression, and identified multiple cytokines and chemokines involved. Using clinical samples from patients with breast or endometrial cancers and those from healthy donors, he provided direct evidence that TAMs possess cancer-type specific transcriptional profiles that are associated with the clinical outcome for patients. Jeff's series of studies shed light on the importance of TAMs as a therapeutic target for malignant solid tumours, which was underestimated in the cancer research field 20 years ago. Due to his pioneering work on TAMs, the elucidation of the tumour-promoting mechanisms of macrophages is now one of the hottest topics in tumour immunology.

Jeff also emphasized translating data from mouse models to human cancers. He actively participated in the European multicentre initiatives COST actions Mye-EUNITER and Mye-InfoBank, which aimed to discover new therapeutic targets for cancer treatment by focusing on myeloid cells. Additionally, he played a crucial role as a founding member, director and advisor of Macomics Ltd, a start-up dedicated to the development of innovative treatments that specifically target TAMs. He also aspired to the further advancement of macrophage biology through interdisciplinary exchange. To that end, Jeff founded a symposium at the University of Edinburgh 10 years ago, called ‘Cells for All Seasons: Macrophages in Health and Disease’. The symposium, now in its seventh edition, has served as an invaluable platform for researchers, post-docs and students actively involved in macrophage biology and offered an opportunity to engage, collaborate, and exchange knowledge with fellow professionals.

Aside from his remarkable research contributions, Jeff stood out for his exceptional qualities as a mentor and leader. He consistently served as a source of inspiration, guidance and unwavering support for everyone in his vicinity. He also fostered an atmosphere of camaraderie and collaboration, encouraging us to think boldly and push the boundaries of scientific exploration. Under his mentorship, we were empowered to pursue our scientific aspirations with confidence, knowing that Jeff had our backs every step of the way. Jeff's unwavering dedication to his lab members went beyond professional obligations. He genuinely cared about our well-being and actively worked towards our success. Whenever his lab members achieved success, Jeff invited us to his gallery-like home adorned with his cherished Japanese artwork, and celebrated with special wine and wonderful dishes prepared by his wife, Dr Ooi-Thye Chong. It mirrored that of a proud parent.

His passing brought deep sorrow to us. But we know Jeff doesn't want us to get sentimental. He would prefer us to remain focused on our scientific pursuits. Respecting Jeff's legacy, we will persist in our endeavours to make meaningful contributions to society by addressing scientific inquiries and advancing knowledge in our respective fields. Thank you, Jeff.

